# Father presence, adolescent girls’ resilience, psychological security, and achievement goal orientation: examining direct and indirect associations

**DOI:** 10.3389/fpsyg.2024.1403403

**Published:** 2024-10-04

**Authors:** Jiayi Zhou, Xueyan Wei, Lingfang Xue

**Affiliations:** School of Humanities, Jiangnan University, Wuxi, China

**Keywords:** father presence, adolescent girls’ resilience, psychological security, achievement goal orientation: examining direct and indirect associations father presence, achievement goal orientation, resilience, mediating effect

## Abstract

In the developmental research, studies on the importance of fathers in the parent–child relationship is insufficient, especially the father-daughter relationship. Thus far, a few studies have investigated whether father presence influences adolescent daughters’ resilience. Therefore, this study investigated the extent to which intermediary factors affect the relationship between father presence and daughters’ resilience. A total of 718 Chinese female high school students participated in a questionnaire survey. The results showed father presence was positively related to resilience in female high school students. Additionally, psychological security fully mediated the relationship between father presence and daughters’ resilience, whereas achievement goal orientation partially mediated this relationship; specifically, the mastery-approach and performance-approach orientations. The results highlight the importance of considering the meaning and implications of father presence and daughter’s resilience in Chinese culture.

## Highlights

- Father presence was positively related to the resilience of Chinese daughters.
- Father presence was indirectly associated with resilience through mediation pathways of psychological security and achievement goal orientation.
- Father presence was indirectly associated with resilience via fully mediating ways such as interpersonal security (a sub-factor of psychological security), mastery goal and performance-approaching goal. (Two sub-factors of achievement goal orientation).

## 1 Introduction

The “Blue Book of National Depression in 2022” (Institute of Depression, 2022) reveals that 95 million people in China are currently battling depression and the prevalence rate of depression among Chinese adolescents has reached 15 to 20%. This figure includes a worrying 30% of individuals under 18 years old, with school students comprising half of this demographic (Mayifan, 2024).

Adolescence, a period of seeking independence and establishing equal footing with parents, is often accompanied by heightened stress due to physiological changes. This stress can lead to depression, a prevalent issue affecting 45% of adolescents.

Family and interpersonal relationships play a crucial role in adolescent mental health. A survey found that 77 and 69% of adolescents with depression struggle in these areas. What’s more, a study of 47 students experiencing depression revealed that 63% reported experiencing harsh/controlling behavior, neglect/lack of care, or conflict/domestic violence within their families. This underscores the detrimental impact of dysfunctional family dynamics on adolescent mental health (Chenweiqi, 2024).

For girls, adolescence presents unique challenges for them, who often experience increased emotional sensitivity and a need for social validation (Jiang et al., 2022). These factors, coupled with the influence of societal norms like son preference in certain regions like Guangxi Province, can create a complex environment where girls are more vulnerable to peer isolation, psychological violence in schools, and emotional neglect or abuse within their families (Yin, 2006; Liang, 2010).

Notably, several studies highlight that the father-daughter bond is not merely significant, but its impact may be particularly profound in certain aspects. For instance, daughters’ closeness with their fathers has been shown to have a profound effect on adult depressive symptoms, marital outcomes, and educational and career achievements (Dmitrieva and Espel, 2023). The absence of paternal emotional support or closeness with fathers has been linked to the emergence and exacerbation of mental health issues and emotional struggles in adolescent daughters (Allgood et al., 2012; Coley, 2003; Demidenko et al., 2015).

Other studies have shown that family-active resources can support the mental health of girls. For example, Wu et al. (2017) and Crockett et al. (1993) investigated a unique family resource, father presence, and it has been described as “his psychological presence in the offspring” (Neufeldt and Guralnik, 1994). Which implies that the father is psychologically close to his child (Krampe, 2009), and found paternal presence was positively associated with children’s resilience. In China, a study found the father-daughter relationship intensified the resilience of adolescent girls in actively coping with risks (Pu et al., 2012). Thus, it is valuable to explore the link between father presence and resilience among adolescent girls.

Resilience is the process by which individuals make use of their internal and external resources in the face of adversity and ultimately achieve good adaptation (Xue, 2019). Strength perspective theory posits that individuals can use their strengths and resources to solve problems, survive adversity, and overcome setbacks (Saleebey, 2004).

Previous research has shown that father presence plays a positive role in children’s resilience, psychological security, achievement goal orientation, and confidence (Pu et al., 2012; Yang and Zhang, 2016). Studies conducted in Western countries indicate that daughters who maintain high-quality relationships with their fathers tend to exhibit greater self-confidence, independence, and achieve higher academic and career success compared to those with poorer paternal relationships (Gordon, 2016; Nielsen, 2014). These underscore the pivotal role of a positive father-daughter bond in fostering positive outcomes in daughters’ lives though those factors.

The literature has addressed how father presence affects children’s resilience. For example, Kağıtçıbaşı and Ataca (2005) argued that fathers satisfy daughters’ psychological needs, including the need for psychological security, for psychological security, Maslow et al. (1945) described psychological security as “a feeling of confidence, security, and freedom from fear and anxiety, particularly when one’s present (and future) needs are met.” Psychological security includes interpersonal security and certainty in control. Interpersonal security means providing individuals more external resources and perceiving more support for people when dealing with questions. The sense of control identified in the psychological security of adolescents is mainly reflected in the sense of control over the future life (Xue, 2019), thus, we can say enhanced psychological security can promote girls’ resilience.

For achievement goal orientation, achievement goal orientation is defined as the perception of one’s learning activities, academic performance, and success goals, including one’s judgment of their abilities, attributions of success or failure, and emotions (Ames, 1992). Additionally, father presence can enhance achievement goals (Liang and Kaisha, 2021). Studies indicated that African American women grasp how the perceived and desired involvement of one’s father can shape their career successes, and aspirations (Stewart, 2014), which promotes children’s resilience. Based on the literature, we were interested in further investigating how father presence impacts resilience among adolescent girls through these key variables.

### 1.1 Father presence and daughters’ resilience

Reciprocal role theory specifically points to the uniqueness of father-daughter relationships and emphasizes the importance of the father in the daughter’s development (Johnson, 1963, 1975). An active role of fathers in the intimate care of their offspring could enhance sons’ perception of relational connectedness and daughters’ feeling of autonomy (Judd et al., 1995). However, fathers’ relative emotional detachment and higher insensitivity to their children’s cues might offer inadequate support to their daughters (Lovas, 2005).

### 1.2 Psychological security as the mediating role

Other lines of research have demonstrated the importance of the father-daughter relationship and this relation can intensify daughters’ psychological security. For example, studies have demonstrated fathers can have an influence on the development of adolescent girls’ personality (Hetherngton, 1972). The father-daughter relationship was identified as a main factor affecting the development of a daughter’s love and work style (Soh, 1993; Tessman, 1988). Gao (2013) used qualitative research methods to analyze the relationship between the parent–child relationship and adolescent depression and identified “indifference” in the father-daughter relationship had a significant impact on depression in daughters. Gender differences are more evident in adolescence compared to the other developmental stages (Heller et al., 2007). Fathers reported more closeness with their daughters than their sons (Cassano et al., 2014), thus, we speculated that there would be a significant association between father presence and adolescent girls’ psychological security.

The father-daughter relationship can be described using attachment theory (Bowlby, 1988), which posts the father-daughter relationship has a profound impact on daughter’s interpersonal security and psychological well-being.

First, research revealed a negative correlation between positive father-daughter attachment and a daughter’s interpersonal challenges (Zia and Ali, 2014). When daughters got a strong attachment to her father, she feels accepted, and demonstrates social interaction, which can greatly intensifies daughters’ interpersonal security. And interpersonal support is an important dimension of resilience, so we can say an active father-daughter attachment can grow girls’ interpersonal security, building their resilience.

Second, daughters who are securely attached, exhibiting low levels of anxiety and avoidance in attachment, engage effectively in conversation with their fathers, displaying care and concern (Akhtar et al., 2019). And daughters feel warmth, safe, and support from their fathers and families, at the same time, family support is an significant dimension of resilience, thus, girls’ resilience can be developed though this way.

So psychological security plays a crucial role. In conclusion, active parental engagement from the father as a primary attachment figure enables the daughter to fulfill her fundamental psychological needs through this relationship, contributing many aspects of her life, like resilience in responding to risks and challenges. Therefore, psychological security could play a mediating role in the relationship between father presence and daughters’ resilience.

### 1.3 Achievement goal orientation as the mediating role

Achievement goal orientation has been found to be a protective factor in fostering individual resilience (Garmezy et al., 1984). There is research evidence that indicates father presence promotes children’s achievement goal orientation. Daughters without highly engaged fathers are more likely to experience poorer academic performance, such as lower IQ scores and substandard school performance (Krohn and Bogan, 2001). Zia et al. (2015) established that the quality of the father-daughter relationship significantly influences a daughter’s academic success among a group of adolescent female participants. Girls who achieve academically are more resilient, as academic success is a significant aspect of achievement goal orientation, and goal orientation is a key dimension of resilience.

According to the Resilience Model (Richardson, 2002), external protective factors, like a strong family support system, play a crucial role in nurturing positive individual qualities in adolescents. By addressing their needs, these factors provide a buffer against adversity and promote healthy development. The study suggests that a high-quality father presence can significantly contribute to adolescent resilience by providing essential external support. This includes material security, emotional support, guidance in planning, and positive role modeling. A supportive father can not only meet adolescents’ external needs but also empower them to pursue their goals with confidence and determination. This process strengthens their internal positive qualities, ultimately equipping them with the resilience needed to navigate challenges and achieve successful adaptation.

Guan (2018) found that achievement goal orientation significantly mediated the relationship between father presence and adolescents’ academic resilience in Chinese high school students.

The research of Li and Lü (2018) consistently demonstrate a significant positive correlation between high achievement goal orientation and resilience in elite athletes. This suggests that individuals who are highly driven by achievement are more likely to possess strong resilience. Individuals with a high achievement goal orientation are characterized by their dedication to tasks and goals, actively seeking resources and strategies to achieve success. This proactive mindset, combined with their perseverance and problem-solving skills, contributes to their enhanced resilience when encountering challenges.

Other research suggested that the conduct of fathers holds immense significance in molding a child’s personality and modifying their behavior through the lens of achievement goal orientation. For instance, female accomplishment and success in male-dominated fields, such as mathematics, heavily rely on the amount of time, direction, and encouragement they receive from their fathers. These proactive and engaged actions can assist girls in overcoming their insecurities, transforming them into confident and resilient individuals who embrace challenges head-on. This, in turn, contributes to building girls’ resilience (Zia et al., 2015). These studies show that father presence, achievement goal orientation, and resilience are positively related. Therefore, achievement goal orientation could play a mediating role in the relationship between father presence and daughters’ resilience. However, the mechanisms and interplay of factors involved in the fathers-daughter relationship that fosters resilience requires further exploration, especially with regard to psychological security and achievement goal orientation.

### 1.4 The present study

The present study sought to explore the relationships between father presence, psychological security, achievement goal orientation, and resilience among adolescent females. In particular, we explored whether father presence, psychological security, and achievement goal orientation would positively predict daughters’ resilience. Lastly, we tested a mediation model in which psychological security and achievement goal orientation were mediators in the relationship between father presence and resilience among adolescent females.

Based on the literature, we hypothesized father presence, psychological security, achievement goal orientation, and resilience would be positively related (hypothesis 1); father presence would positively predict daughter’s resilience (hypothesis 2); psychological security would mediate the association between father presence and daughters’ resilience (hypothesis 3); and achievement goal orientation would mediate the association between father presence and daughters’ resilience (hypothesis 4).

## 2 Materials and methods

### 2.1 Participants

This study selected a total of 718 female students from four schools, including two Middle schools and two Senior high schools in Jiangsu and Shanxi provinces in China for a random sampling questionnaire survey. All high schools are public. The adolescents’ age was from 13 to 18, and they are from grades 7th to grades 12th. After collecting 51 invalid questionnaires, 667 valid questionnaires were obtained. The effective response rate of this questionnaire survey was 92.9%. The Demography distribution of the subjects is shown in Table 1.

**Table 1:** Distribution of demography variables of subjects (*N* = 718).

Demographic variables	Category	Number	Precentage (%)
School type	Middle School Student	353	49.2
	Senior High School Student	365	50.8
Territory	City	424	59.1
	Rural area	294	40.9
Only child or not	Yes	386	53.8
	No	332	46.2

### 2.2 Procedures

Participants’ parents, classroom teachers of the 7th to 12th grades were contacted to complete this survey. Students performed their cognitions of their father presence, psychological security, achievement goal orientation and resilience. We arranged a questionnaire survey in the classroom to ensure the following two points. On the one hand, it reduces the possibility of parents interfering with students and directly answering instead of students; On the other hand, every student can complete this off-line paper task. All participants were sent gifts value 50 yuan.

### 2.3 Measurement

#### 2.3.1 Father presence

This study intends to use the “Father Presence Questionnaire” derived from Krampe and Newton (2006), Father presence was revised into the Chinese version (Xue, 2019). In this study, this measure was high internal reliability (α = 0.870). This study intends to use the self-developed “Adolescents’ Father Presence Scale,” which consists of 38 items and includes 7 dimensions: material security, emotional care, participation and guidance, future planning, experience transmission, role model demonstration, and overall intention. This scale adopts the Likert five point scoring method, 1–5 represents completely no compliant ‘to’ completely compliant, the higher the score means the higher the degree of father presence of teenagers. In the study, the exploratory factor analysis result of the scale was: KMO = 0.971, with a cumulative explanation rate of 74.70%. The results of confirmatory factor analysis are: χ2/df = 3.343, GFI = 0.871, AGFI = 0.818, NFI = 0.914, IFI = 0.947, TLI = 0.920, CFI = 0.918, RMSEA = 0.078. The total α is 0.878, and the reliability of each sub dimension is 0.871, 0.906, 0.857, 0.901, 0.928, 0.899, and 0.937, respectively. The above results indicate that the scale has good reliability and validity, and is suitable for this study.

#### 2.3.2 Psychological security

This study intends to use the “Security Questionnaire” (SQ) developed by Cong and An (2004), which consists of 16 items and includes two dimensions: interpersonal security factor and deterministic control factor. This scale adopts the method of Likert five points scoring, with 1–5 representing “never” to “always,” which includes reverse scoring questions. After recording the reverse scoring question, the higher the total score, the higher the level of psychological security of adolescents. In this study, the exploratory factor analysis result of the scale was: KMO = 0.930, with a cumulative explanation rate of 52.13%. The results of confirmatory factor analysis are: χ^2^/df = 3.402, GFI = 0.908, AGFI = 0.891, NFI = 0.927, IFI = 0.918, TLI = 0.929, CFI = 0.945, RMSEA = 0.008. The overall α coefficient is 0.921, and the reliability of each sub dimension is 0.851 and 0.863, respectively. This indicates that the scale has good reliability and validity, and is suitable for this study.

#### 2.3.3 Achievement goal orientation

This study revised the Achievement Goal Orientation Questionnaire prepared by Vandewalle (2001), which has 16 items in total, including three dimensions of Mastery learning goal, achievement approach goal, and achievement avoidance goal. This scale adopts the Likert Quintuple method, with 1–5 representing “never” to “always.” The higher the score, the stronger the adolescent’s achievement goal orientation. It was found that the load of two projects exceeded two dimensions and was therefore deleted. Exploratory factor analysis of the 14 retained items showed that KMO = 0.902, with a cumulative explanation rate of 70.29%. Confirmatory factor analysis’ conclusions are: χ^2^/df = 4.018, GFI = 0.932, AGFI = 0.921, NFI = 0.910, IFI = 0.920, TLI = 0.917, CFI = 0.901, RMSEA = 0.080. The overall Cronbach α is 0.899, and the reliability of each sub dimension is 0.880, 0.908, and 0.787, respectively. This indicates that the scale has good reliability and validity, and is suitable for this study.

#### 2.3.4 Resilience

This study intends to use the “Adolescent Psychological Resilience Scale” developed by Hu and Gan (2008). The scale consists of 27 items, including two high-order factors (physical and social support), and five sub dimensions (goal focus, emotional control, positive cognition, family support, and interpersonal assistance). This scale adopts the Likert five points rate, with 1–5 representing “completely inconsistent” to “completely consistent.” After recording the reverse scoring question, the higher the total score, the stronger the individual’s resilience. In this study, the exploratory factor analysis result of the scale was: KMO = 0.860, with a cumulative explanation rate of 55.78%. The results of confirmatory factor analysis are: χ^2^/df = 2.967, GFI = 0.891, AGFI = 0.891, NFI = 0.860, IFI = 0.910, TLI = 0.890, CFI = 0.901, RMSEA = 0.051. The α coefficient is 0.880, with each subdimension were 0.901, 0.860, 0.819, 0.792, and 0.889, respectively. This indicates that the scale has good reliability and validity, and is suitable for this study.

### 2.4 Data analysis

Data analyses were performed using SPSS21.0, AMOS17.0, MPLUS7.0 in the following order. We use SPSS21.0 to perform exploring factor analysis, descriptive analysis, correlation analysis and regression analysis; Confirming factor analysis of the scale was confirmed by Amos 24.0; The measurement of the mediator of psychological security and achievement goal orientation between father presence and daughters’ resilience was used by Mplus 7.0.

### 2.5 Common method variance

This study tested common method variance bias with one single-factor test (Williams and McGonagle, 2016). The result illustrated that there was 12% of the total variance, less than the 25% suggesting that there was no covariance between these variables.

## 3 Results

### 3.1 Descriptive statistics and correlation analysis

In order to verify the relations among variables, we conducted Descriptive Statistics and Correlation Analysis, Table 2 showed the results as follows (see Table 2).

**Table 2:** Descriptive statistics and bivariate correlations between the main variable (*N* = 718).

Variable	Mean	SD	Father presence	Achievement goal orientation	Psychological security	Resilience
Father presence	146.12	32.29	1			
Achievement goal orientation	52.83	10.08	0.331***	1		
Psychological security	53.08	13.72	0.502***	−0.048	1	
Resilience	95.99	16.76	0.296**	0.164**	0.629***	1

*Represents *p* < 0.05, **represents *p* < 0.01, ***represents *p* < 0.001.

Table 2 presented studied variables among means, standard deviations, and correlations. Hypothesis 1 was verified. Father presence had a positive relation with psychological security, achievement goal orientation and resilience. Higher father presence, stronger psychological security and stronger achievement goal orientation had a higher association with children’s resilience. Also, there is the strongest relations between psychological security and the resilience (*r* = 0.629***, *p* < 0.001).

### 3.2 Analysis of regression

Our study took father presence and psychological security, achievement goal orientation as independent variables and resilience as dependent variables so as to further test predicting effect of father presence and psychological security, achievement goal orientation on resilience of middle school female students (grades 7–12), the results are shown in Table 3.

**Table 3:** Regression analysis of variables predicting resilience (*N* = 718).

Dependent variable	Independent variable	*R*	*R* ^2^	△*R*^2^	SE	*β* 值	*t* 值	*F* 值
Resilience	Psychological security	0.631	0.398	0.398	0.035	0.512	17.734***	400.66
	Father presence	0.756	0.572	0.174	0.021	0.132	4.78**	50.07
	Achievement goal orientation	0.762	0.581	0.009	0.031	0.021	4.087*	21.765

*p < 0.05, **p < 0.01, **p < 0.001, the same below.

As Table 3 shows, all three variables entered the regression equation, contributing a cumulative explanation rate of 58.1%, with psychological security having the strongest predictive effect, with an explanation rate of 39.8% (*β* = 0.512, *t* = 17.734***). With an explanation rate of 17.4% for father presence (*β* = 0.132, *t* = 4.78**), with an explanation rate of 0.9% for achievement goal orientation (*β* = 0.021, *t* = 4.087*), and the *F* values of all four models reached a significant level. Hypothesis 2 was supported.

### 3.3 Mediating effect analysis

**Figure 1:**
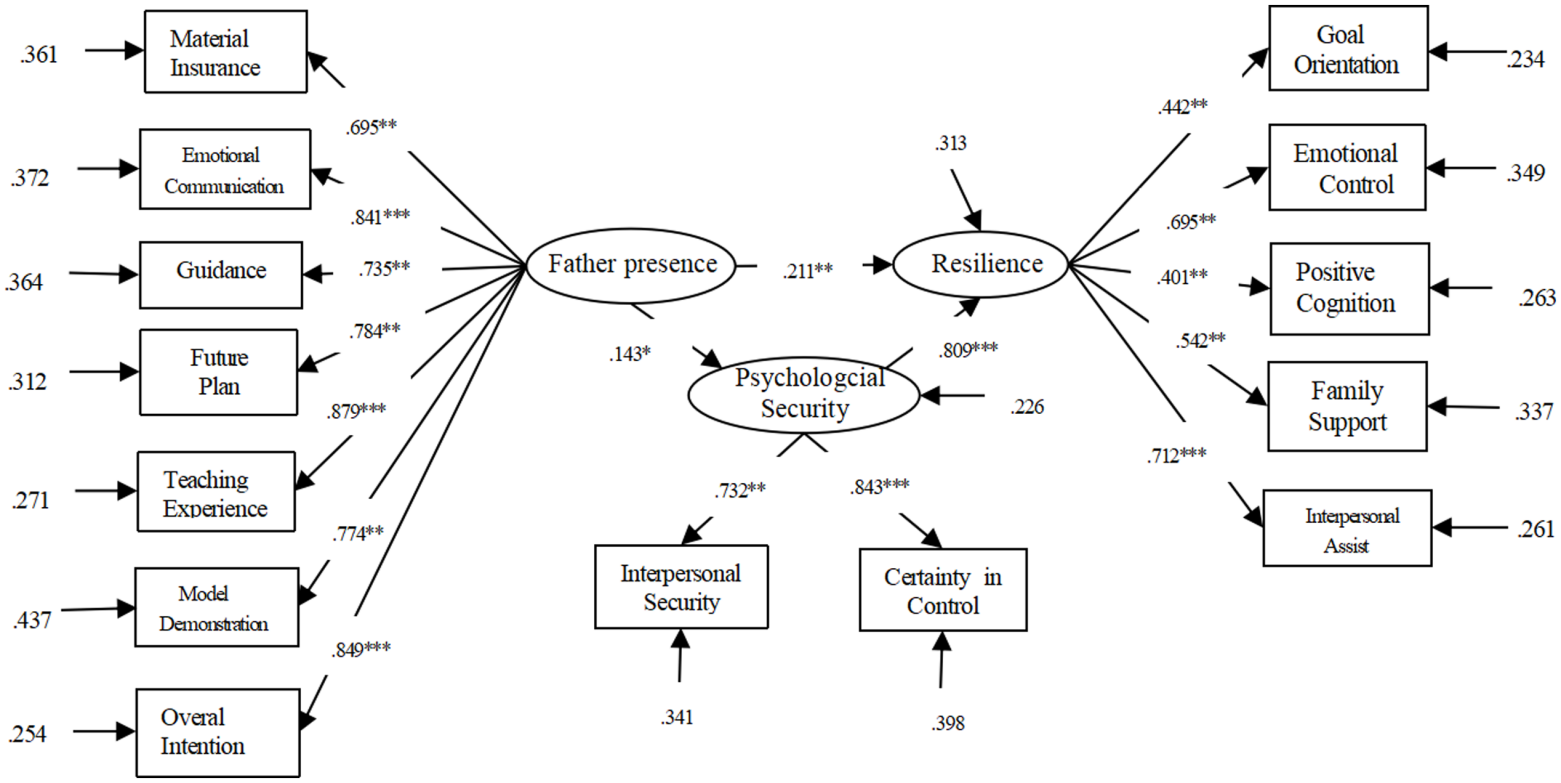
Mediation model diagram of psychological safety between father presence and resilience.

This article used Mplus7.0 software for mediation analysis. We considered father presence as the independent variable, daughters’ resilience as the dependent variable, and psychological security and achievement goal orientation as the mediating variable. We checked the following mediating effect (Figure 1).

### 3.4 Mediating effect analysis of psychological security

**Figure 2:**
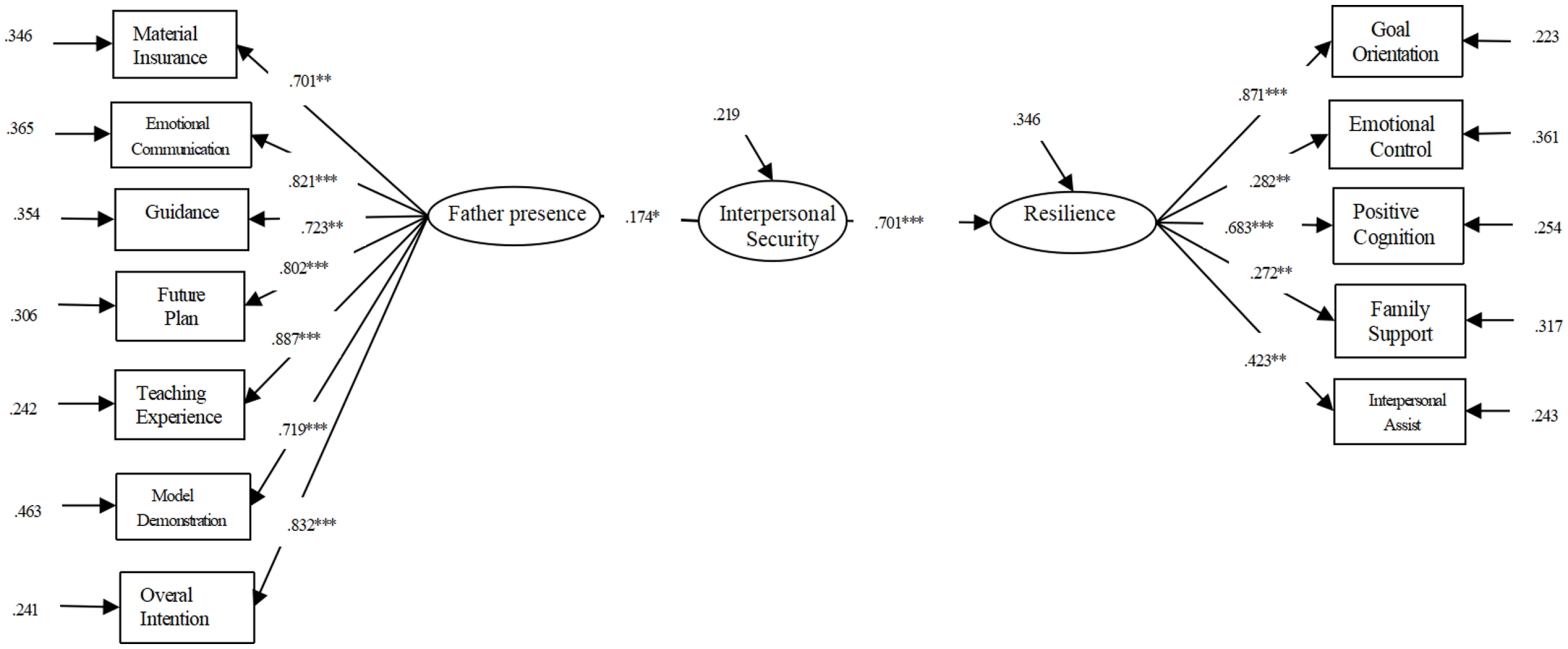
Mediation model diagram of different dimensions of psychological safety between father presence and resilience.

The analysis conclusion was presented in Table 4, χ^2^/df = 3.710, RMSEA = 0.071, CFI = 0.926, TLI = 0.908, SRMR = 0.056. This set of data manifested that psychological security mediated function. The mediating effect was 1 (Figure 2), which also supported hypothesis 3.

**Table 4:** Mediating effect of psychological security between father’s presence and resilience (*N* = 718).

χ^2^/df	RMSEA	CFI	TLI	SRMR
3.710	0.071	0.926	0.908	0.056

### 3.5 Mediating effect analysis of achievement goal orientation

**Figure 3:**
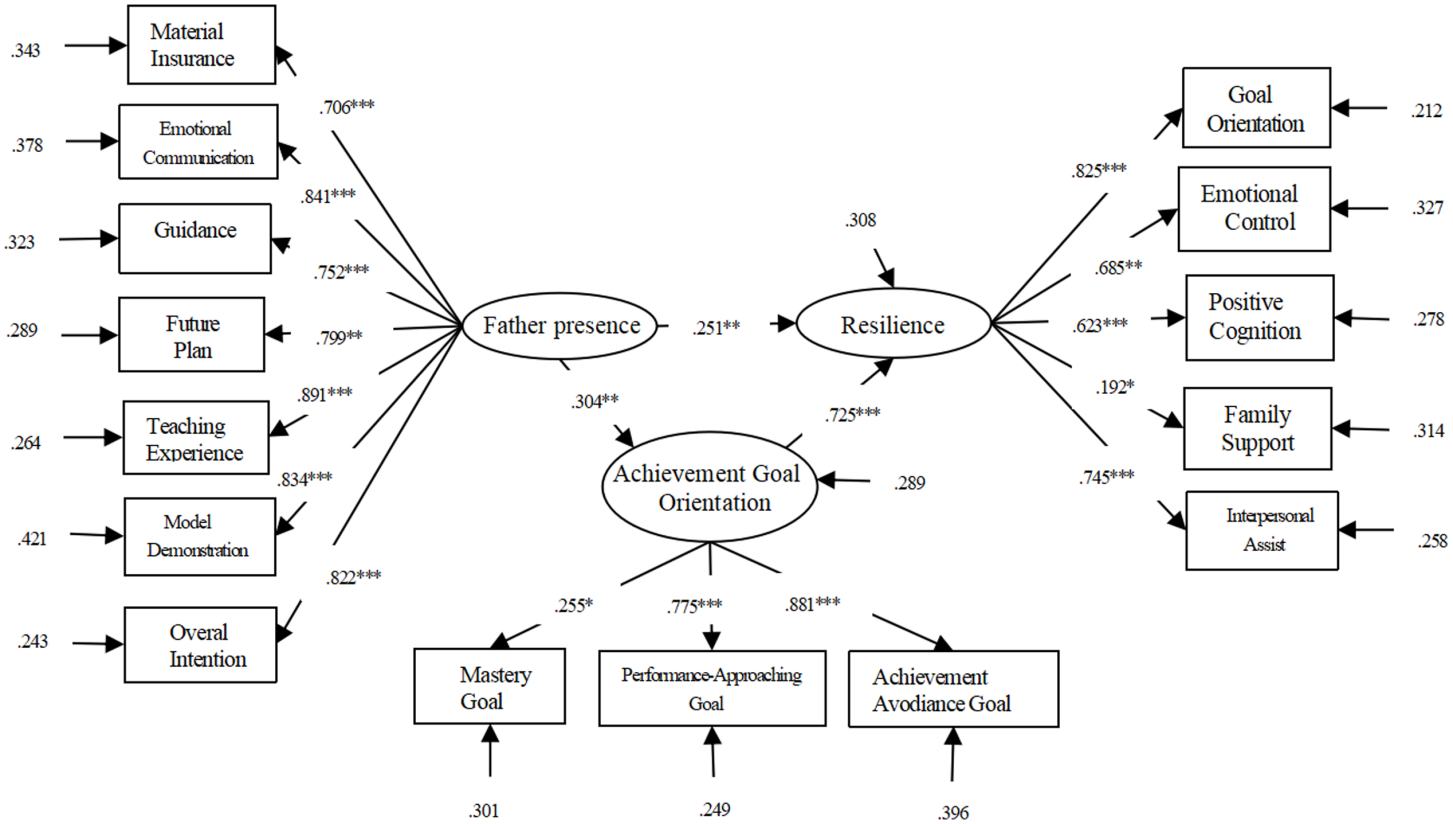
Mediation model diagram of achievement goal orientation between father presence and resilience.

The mediating effect displayed in Table 5, χ^2^/df = 4.110 <5, RMSEA = 0.078 < 0.08 CFI = 0.941, TLI = 0.901, SRMR = 0.072 < 0.08. The statistic from this study proclaimed that achievement goal orientation played a partial mediating role between the father presence and students’ resilience. The mediating effect was 0.526 (Figure 3). Assumption 4 was supported.

**Table 5:** Analysis of the Mediating effect of achievement goal orientation between father presence and resilience (*N* = 718).

χ^2^/df	RMSEA	CFI	TLI	SRMR
4.110	0.078	0.941	0.901	0.072

In this study, we conducted a Bootstrap test with a confidence interval of 96% and 5,000 repeated samples. The results show (see Table 6) that in the path of father presence – psychological security – resilience, the 96% confidence interval is [0.007, 0.052], and the confidence interval does not include 0, which suggested that psychological security has a significant intermediary effect between father presence and resilience. Considering that different dimensions of psychological security may have different effects on the whole, this study uses two factors of psychological security as mediating variables. The results show that the fitting index of the model is: χ^2^/df = 6.436, RMSEA = 0.121, CFI = 0.841, TLI = 0.790, SRMR = 0.098. The mediating path for determining the certain in control is not significant, so deleting this path yields a fitting index of: χ^2^/df = 5.098, RMSEA = 0.069, CFI = 0.917, TLI = 0.899, SRMR = 0.060. In the revised model, the coefficients of each path reached a significance level (see Figure 2). The interpersonal security plays a complete mediating role between the father’s presence and resilience, that is, the father’s presence has a complete impact on the resilience of adolescents through interpersonal security.

**Table 6:** Mediating effect of psychological security between father’s presence and resilience (*N* = 718).

Path effect	95% confidence interval		Indirect effect value
	Boot CI upper limit	Boot CI lower limit	
Father presence-psychological security-resilience	0.007	0.052	0.118
Father presence-Interpersonal security-resilience	0.026	0.078	0.156

**Figure 4:**
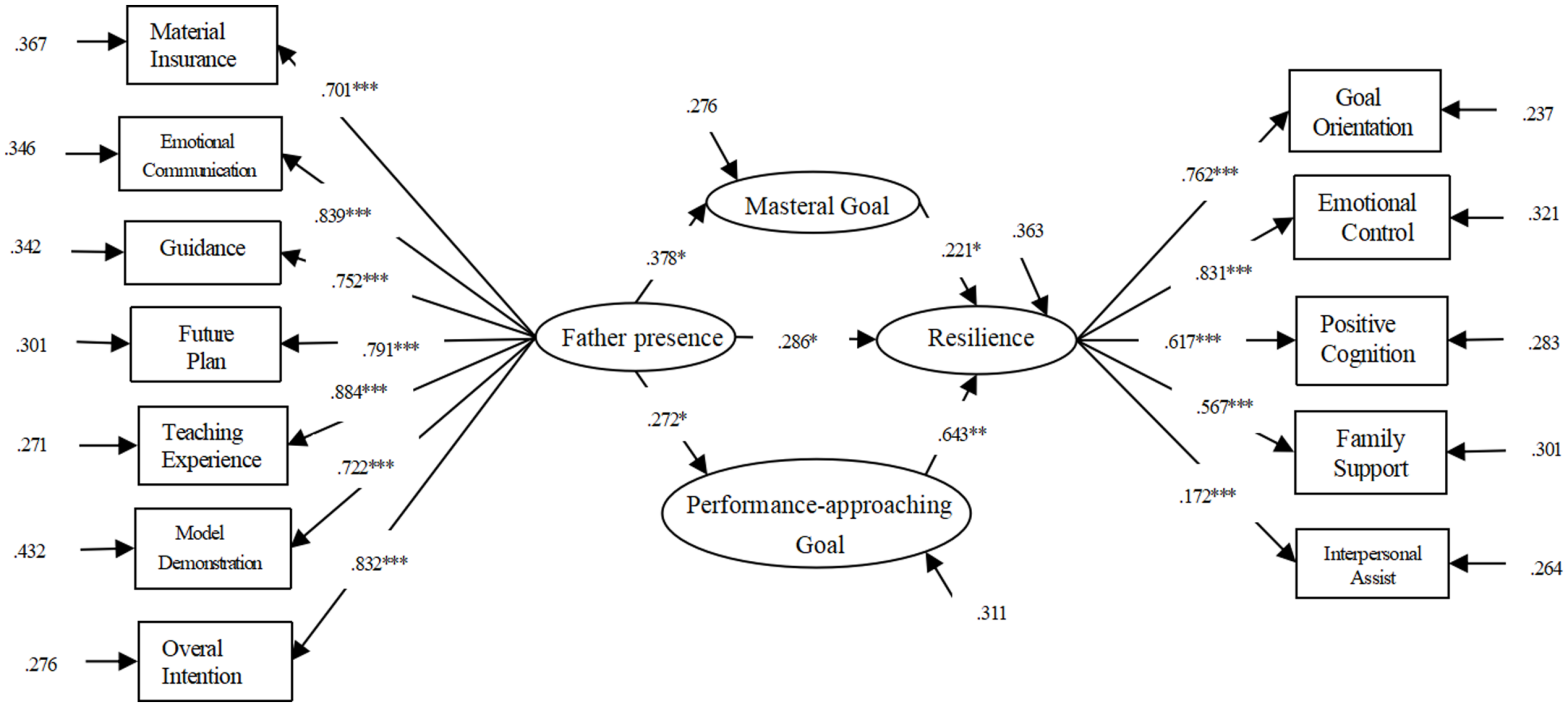
Mediation model diagram of different dimensions of achievement goal orientation between father presence and resilience.

In the path of father presence-interpersonal security-resilience, 96% of the Bootstrap confidence interval is [0.026, 0.078], and 0 was not included by the confidence interval, indicating that interpersonal security has a significant intermediary effect between the correlation of father presence and students’ resilience (see Table 6). The results show (see Table 3) that in the father presence-achievement goal orientation-resilience, its 96% confidence interval is [0.094, 0.169], and 0 wasn’t involved, indicating that achievement goal orientation has a significant intermediary effect between father presence and daughters’ resilience. The results show that the fitting index is: χ^2^/df = 7.127, RMSEA = 0.151, CFI = 0.832, TLI = 0.778, SRMR = 0.101. The mediating path of achievement avoidance goals is not significant, so deleting this path yields a fitting index of: χ^2^/df = 4.209, RMSEA = 0.061, CFI = 0.908, TLI = 0.899, SRMR = 0.054. In the revised model, the coefficients of each path reached a significance level (see Figure 3). That is, Mastery goal and performance approaching goal play a partial mediating role between the father’s presence and resilience. In the path of father presence-Mastery goal-resilience, its 95% confidence interval is [0.021, 0.069], and the confidence interval does not include 0, indicating that Mastery goal has a significant intermediary effect (see Table 7); In the path of father presence – performance-approaching goal – resilience, the 95% confidence interval is [0.040, 0.009], and the confidence interval does not contain 0, which showed that the performance-approaching goal has a significant intermediary effect between father presence and resilience (see Table 7 and Figure 4).

**Table 7:** Mediating effect of achievement goal orientation between father presence and resilience (*N* = 718).

Path effect	95% confidence interval		Indirect effect value
	Boot CI upper limit	Boot CI lower limit	
Father presence-achievement goal orientation-resilience	0.094	0.169	0.24
Father presence-mastery goal-resilience	0.021	0.069	0.09
Father presence-performance-approach goal-resilience	0.040	0.009	0.172

## 4 Discussion

We tested several hypotheses derived from reciprocal role theory (Johnson, 1963, 1975), attachment theory (Bowlby, 1988), and by analyzing the relationship between father presence and daughters’ resilience and the serial mediation of psychological security and achievement goal orientation.

First, father presence, psychological security, achievement goal orientation and resilience would be positively related. Second, father presence was positively related to daughters’ resilience, which provided support for hypothesis 2. Reciprocal role theory specifically points to the uniqueness of the father-daughter relationship and maintains that fathers play a key role in daughters’ development (Johnson, 1963, 1975). This result validates the theory in the Eastern context of China. The length and quality of father care have been found to have a strong positive correlation with the adaptability of a child’s life (Adam et al., 2004). Positive parent–child communication methods can greatly reduce children’s negative social adaptability (Hwang and Lamb, 1997). Qi et al. (2008) found that Chinese parents’ positive parenting styles are conducive to children having positive coping styles when faced with adversity and setbacks. Overall, father presence as an exterior-securing resource could contribute to daughters’ resilience. Daughters will be stronger and braver with the help of a positive parenting style when making decisions and taking action, which is beneficial to daughters’ resilience.

Third, we can find that psychological security was the strongest predictor of daughters’ resilience. Previous evidence suggested that the father-daughter relationship was directly related to adolescent girls’ psychological outcomes, namely, in a non-Western culture. The results in the current study are consistent with this assertion. The father-daughter relationship is positively related to adolescent girls’ through fathers fulfilling their basic needs. Meanwhile, given girls’ higher vulnerability to psychological distress, as represented in a study showing 26% of adolescent girls vs. 15% of adolescent boys in Australia were found to have psychological problems, and that fathers potentially exert more influence on their daughters than mothers (Lawrence et al., 2015; Papini et al., 1991), the psychological security girls experience may in part be due to their relationship with their father, which makes them more resilient. Therefore, we can conclude that father-daughter relationship is good for daughters’ psychological security, which is good for their well-being or resilience. Psychological security mediated the association between father presence and daughters’ resilience, which provided support for hypothesis 3. Furthermore, we found that interpersonal security exerts a total mediator. Consistent with attachment theory, father presence plays an important role in daughters’ psychological security, and adolescent girls have more interpersonal relationships with their fathers; thus, father presence strongly enhances daughters’ interpersonal security. Another explanation for this result could be that adolescent daughters tend to establish interpersonal relationships passively, and to some extent, daughters are more likely to feel the influence of their father’s interpersonal power on them. Favorable behaviors can be realized through sound personal relationships and contribute to resilience. However, only when father presence has a positive effect on the daughter’s sense of security will his presence have an effect on the development of resilience. Based on attachment theory, we identified the mediating mechanism of psychological security by empirical study.

Fourth, achievement goal orientation mediated the association between father presence and daughters’ resilience, providing support for hypothesis 4. Chinese father presence indirectly affected daughters’ resilience through two dimensions of achievement goal orientation, namely, mastery-approach and performance-approach goal orientations. According to self-determination theory (Deci, 1975), human beings’ three basic psychological needs include competence, autonomy, and relatedness. These needs stimulate and sustain the individual’s intrinsic motivation. Father presence can satisfy these needs in daughters, and especially satisfying competence needs could contribute to their inner strength and resilience. Father involvement can contribute to daughters’ competence through mastering tasks and having them demonstrate skills and abilities, thereby contributing to resilience indirectly through satisfying the basic psychological need of competence.

Another great contribution of this study suggests that strong parental attachments act as a protective factor against depression in adolescents, aligning with attachment theory’s emphasis on the importance of supportive relationships for psychological wellbeing.

This study suggests that Chinese parental involvement can positively influence adolescent development by fostering their engagement in school, both behaviorally and emotionally. This aligns with self-determination theory and existing research highlighting the crucial role of parents in shaping academic and emotional growth (Spera, 2005). By promoting positive emotional behaviors, parental involvement can contribute to school success and ultimately improve mental health (Masten et al., 2005). This research aims to further investigate the direct and indirect effects of parental involvement on school engagement, academic achievement, and depression in adolescents.

The strength of study is highlighting the significant impact of father presence on the resilience of Chinese adolescents. Our findings show that high-quality father-child relationships contribute to positive psychological development, especially for girls, where psychological security plays a crucial mediating role. These results confirm the vital role of fathers as external support for Chinese adolescents, offering valuable insights for family education.

In conclusion, psychological security and achievement goal orientation, both as daughter’s “inner soft power” intensified by the “outer hard power” from father presence, can promote daughters’ resilience. The father can potentially serve as an impressive example in her life, and she tries to view the world according to that perspective. The quality of the time fathers and daughters spend together is essential at all stages throughout the daughter’s life. In particular, fathers’ presence can influence the development of resilience in their daughters through fathers providing daughters with a sense of security and competence. Furthermore, this study, in addition to exploring attachment theory and reciprocal role theory, also draws upon the dynamic equilibrium model of resilience, the dynamic model of father presence, empirical learning theory, and the strength perspective as theoretical frameworks.

### 4.1 Limitations and future research

This study tested a theoretical model associating father presence with psychological security, achievement goal orientation, and daughters’ resilience in a relatively large sample of Chinese adolescents. There are some limitations to be considered when interpreting the findings. First, this was a cross-sectional study; therefore, causal relationships between the variables could not be determined. There are dynamic interactions between the variables (e.g., Davidov and Grusec, 2006; Rubin et al., 1999) such that the resilience of children can also possibly affect parenting behavior. Therefore, future research could test the bidirectional relationships through a longitudinal research design. Additionally, the family structure was not assessed, which could contribute to the relationships found. For example, on the one hand, it has been reported that children from single-parent families have a greater probability of having lower resilience levels. On the other hand, the single-parent family structure automatically determines the contact ratio between the child and father or mother. In a sample of children from single-parent families, the proportion of father presence is prone to differ from that of dual-parent families. As such, the results should be interpreted with caution. Researchers should try to ensure the sameness of family structure in future research, so that better conclusions can be reached. Third, we can expand participant diversity including a wider range of ethnicities, socioeconomic backgrounds, ages, and geographic locations. Fourth, the replication can from multiple perspectives like incorporating input from parents (on their attachment styles) and teachers (on academic performance) to provide a more comprehensive understanding. Fifth, we did not explore the role of mothers and the unique contributions of mothers to adolescent resilience and comparing them to the role of fathers.

In future research, we would like to incorporate analyses of the study variables across different categories of adolescents, including analyses of differences based on many factors like student type (e.g., public vs. private school, rural vs. urban), student gender (e.g., male vs. female), family structure (e.g., single-parent vs. two-parent households), parenting style (e.g., authoritative vs. permissive), father’s education level (e.g., high school diploma vs. college degree).

In this respect, future researchers may benefit from obtaining aggregated information from other sources of parental attitudes and parent–child relationships. Furthermore, parenting style should be considered. Resilience can be positively influenced by parenting style (Kritzas and Grobler, 2005; Zakeri et al., 2010). Another limitation of this study is the homogeneity of the sample, which limits the generalizability of the results. Therefore, in subsequent studies, researchers should expand the sampling range and increase the sample size so as to improve the representativeness of the sample. For example, the social class of the family, the extent of family interaction, and urban–rural differences should be considered. Lastly, we investigated the mediating role of psychological security and achievement goal orientation in the relationship between father presence and adolescent girls’ psychological resilience. However, other possible mediating variables are worth exploring, such as self-control ability, parental pressure, and family adaptability (Bush et al., 2017; Malia, 2006). Therefore, future research will deepen our understanding of the mechanisms involved in how the father-daughter relationship contributes to daughters’ resilience.

### 4.2 Practical implications and practical policies

Our findings provide several practical implications for the development of resilience in girls. The study revealed that father presence, psychological security, and achievement goal orientation contributed to resilience in female high school students in mainland China. However, father presence had both an indirect and direct association with resilience. These findings have practical implications for fathers. First, a father’s presence is important for the daughter’s psychosocial development (and resilience in particular). Our results support the expanded ecological model of father involvement (Cabrera et al., 2014), which emphasizes fathers’ unique role in the family system. Therefore, the results of this study have utility for informing effective parenting education interventions. For example, fathers may need help in strengthening communication, expressing their concerns, and expressing empathy to their daughters, so that their daughters psychological needs for competence can be met or satisfied, which can contribute to the development of resilience.

Father presence as a protective factor is important for girls as they enter the critical stage of adolescence; that is, girls’ mental health is positively affected by having a good father-daughter relationship. Furthermore, policies should be considered to help promote resilience. Taking the propaganda policy as an example, it would be important for fathers to understand the significance of father presence, take relevant education courses, strengthen the construction of community cooperation, and accept family guidance and treatment. In addition, more attention is needed toward solving problems inside the family as well as outside the family. From within the family, fathers need to provide a supportive environment for their children’s growth. However, it should be noted that the family environment is affected to some extent by the unbalanced factors of the social structure. Therefore, there is a need to establish social equality, and guarantee and strengthen the rights to education and the welfare of teenagers and children. From the perspective of external factors of the family, we found that fathers foster the development and stability of adolescent girls; however, sharing the role of parenting may have a more positive impact on the maturity of the family and children (Cabrera et al., 2014; Choi and Becher, 2019).

Meanwhile, the training of family education teachers and workers should be increased, and an open, inclusive social family education atmosphere needs to be created. Both the home and school environments have an effect on the development of children’s resilience. For example, Liu et al. (2022) suggested that school-adjusted outcomes can be improved by elevating children’s resilience. In schools, educators can help teenagers determine their long-term goals and personal plans by offering psychoeducational activities, such as lectures on psychological and career development, to facilitate students’ psychological security and achievement goal orientation, which would contribute to the development of resilience in adolescent females.

Society recognizes the vital role of role models, especially those who have navigated similar challenges during adolescence, in promoting resilience among young people. The importance of role models in fostering resilience is supported by established psychological and social science theories, such as social learning and modeling (Bandura and Walters, 1977; McAlister et al., 2008), which emphasize the power of observation and imitation.

For children exposed to adversity, social support plays a crucial role in buffering the stress associated with these experiences, thereby reducing the risk of developing and maintaining mental health problems (Nelson et al., 2020; Pinto et al., 2021).

We also speculate that we could focus on educational practices like enhancing teachers’ resilience and school’s culture of resilience, and social practices like exploring the role of community-based programs in fostering girls’ resilience, including programs that address gender-based violence, promote leadership skills, and provide access to resources (LoVette et al., 2023).

For another, from the perspective of females themselves, we infer that we could focuses on the factors that contribute to resilience in girls facing adversity, including protective factors like supportive relationships, coping skills, and positive self-beliefs (Wambua et al., 2024).

## Data Availability

The datasets presented in this study can be found in online repositories. The names of the repository/repositories and accession number(s) can be found in the article/supplementary material.
